# Gene Ontology-Based Comparative Transcriptomics Provides Evidence of Genetic Adaptations in the *Rhinolophus macrotis* Group (Chiroptera: Rhinolophidae)

**DOI:** 10.3390/ani16152331

**Published:** 2026-07-30

**Authors:** Lin Zhang, Keping Sun, Wentao Dai, Tong Liu, Aoqiang Li, Jiang Feng

**Affiliations:** 1 Jilin Provincial Key Laboratory of Animal Resource and Ecological Security, Northeast Normal University, Changchun 130117, China; zhangl309@nenu.edu.cn; 2 School of Sciences, Beihua University, Jilin City 132013, China; 3 College of Life Science, Jilin Agricultural University, Changchun 130118, China; 4 School of Life Sciences, Central China Normal University, Wuhan 430079, China

**Keywords:** *Rhinolophus*, echolocation, transcriptomics, adaptation evolution

## Abstract

Understanding adaptive evolution has long fascinated evolutionary biologists. Adaptive phenotypic divergence is often driven by modifications to protein-coding sequences. The *Rhinolophus macrotis* group exhibits relatively lower echolocation frequencies relative to body size compared with other rhinolophids, implying distinct evolutionary trajectories. Transcriptomes bridge genotypes and phenotypes. Here, we sequenced brain, liver and cochlea transcriptomes from one individual per species representing five taxa of the *macrotis* group. We performed comparative transcriptomic analyses and detected signals of positive selection. Seven hearing-related genes (*CRYM*, *FOXM1*, *MAP6*, *PYCARD*, *SLC35A2*, *WRB* and *SPRY2*) were under positive selection. Unexpectedly, we also identified five vision-associated positively selected genes (*ARRDC3*, *LZTFL1*, *RAB8A*, *IGFBPL1* and *TRNT1*) in taxa with relatively lower echolocation frequencies within the *macrotis* group, indicating selection on sensory genes. Furthermore, candidate positively selected genes were significantly enriched in metabolism-related GO terms such as catalytic and oxidoreductase activity. Our study offers valuable transcriptomic resources for unraveling adaptive genetic mechanisms in horseshoe bats.

## Simple Summary

Horseshoe bats of the *Rhinolophus macrotis* group emit echolocation frequencies lower than expected given their body size, but the genetic mechanisms behind this distinctive acoustic adaptation remain unclear. We sequenced transcriptomes of the brain, liver and cochlea from five species within the *macrotis* group and the closely related species *R. pusillus*, and performed comparative evolutionary analysis to screen genes under natural selection. We identified 141 positively selected genes across the *macrotis* species, including seven hearing-related genes regulating cochlear development and auditory function, and five vision-associated genes found in species with relatively lower echolocation frequencies within the *macrotis* group. Genes under positive selection were significantly enriched in energy metabolism pathways, which support the high energy demands of bat flight and echolocation. Our results prove that natural selection on sensory and metabolic genes may contribute to the unusual acoustic phenotype of the *macrotis* group. This study provides a new molecular insight into sensory adaptive evolution in horseshoe bats, and offers a foundation for future functional verification of candidate adaptive genes.

## 1. Introduction

Ever since the book “On the Origin of Species by Means of Natural Selection” was published by Charles Darwin 160 years ago [1], adaptive evolution has become an important research question [2]. Understanding the driving forces of speciation processes is a primary goal of evolutionary biology [3,4]. Moreover, inferring the patterns and processes underlying the emergence of phenotypic diversity and new species is a primary goal of evolutionary biology [5]. The development of high-throughput sequencing technology provided more genetic information of evolutionary history [6,7], adaptive traits [8,9,10], and the evolution of phenotypic diversity and speciation [5]. Thereinto, transcriptome analysis has been widely used in studies to reveal the species evolutionary mechanisms [11,12,13,14,15]. Comparative evolutionary approaches can address whether interspecific differences are the result of neutral divergence over evolutionary history or whether they are the result of nonrandom processes, such as adaptation to different environmental conditions [16]. Thus, comparative transcriptomics was usually used to study the genetic basis of evolutionary differences among species.

Over long evolutionary timescales, continuous shifts in extrinsic and intrinsic selective pressures drive natural selection, enabling distinct bat lineages to evolve unique adaptive strategies to diverse habitats [17]. Evolution proceeds via genetic and protein sequence divergence that ultimately shapes organismal phenotypes [18,19,20]. The changes in phenotype can reflect the changes in genes. Fundamentally, novel phenotypic traits in bats originate from molecular alterations in genetic material that are filtered and retained under sustained selective pressures [21,22].

Echolocation is a complex, defining sensory trait of bats, involving the generation, reception, and neural processing of ultrasonic pulses for navigation, obstacle avoidance, and hunting [23,24]. Rhinolophid bats possess the most sophisticated echolocation systems [25], and the positive selection of auditory genes in the study of genome data was detected [26]. It revealed the adaptation of auditory sensory perception in rhinolophid bat lineages [26,27,28]. Current studies on the evolution of bat acoustics have been conducted at the population level or in bats with different echolocation types; the molecular mechanisms underlying acoustic differences between closely related bat species are rare. Horseshoe bats (Rhinolophidae) possess specialized constant-frequency (CF) ultrasonic echolocation, distinct from the frequency-modulated (FM) calls of most other bats [25]. Long CF signals enable Doppler-shift compensation for detecting fluttering prey, supported by derived morphological traits including elaborate noseleaves, enlarged nasal capsules and pinnae that mediate call emission and echo reception [29]. In general, forearm length exhibits a negative correlation with the dominant frequency of echolocation calls in bats [30].

**Figure 1:**
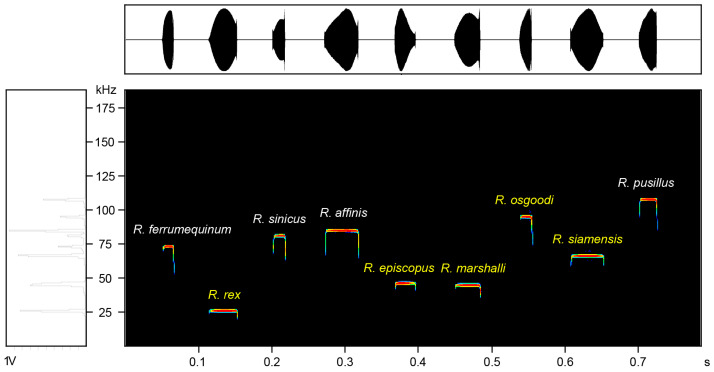
Echolocation calls emitted by different horseshoe bat species, arranged in descending order of body size. Species marked in bright yellow belong to the *macrotis* group.

The *Rhinolophus macrotis* group is a specific species group among the genus *Rhinolophus*. The *macrotis* group comprises multiple closely related taxa. The focal clade distributed across China and adjacent Southeast Asia includes six species, namely *R. episcopus*, *R. siamensis*, *R. rex*, *R. osgoodi*, *R. marshalli*, and *R. schnitzleri*. Among them, *R. schnitzleri* was not sampled owing to its extremely narrow distribution, and suitable tissue samples could not be collected during fieldwork. In addition, the *macrotis* group also incorporates *R. macrotis* (distributed across Nepal) and the recently described *R. kirghizorum* from Central Asia, which are geographically remote and also absent from our sampling [31,32,33]. These species are closely related species, and experienced recent and rapid diversification during the Pleistocene [31]. Compared with other *Rhinolophus* species, echolocation frequencies for the species within the *macrotis* group exhibit differences, except in *R. osgoodi*, which were lower relative to their body size [34] (Figure 1). Although some acoustic-related genes exhibit sequence convergence in echolocating bats [35,36,37,38,39], different echolocation traits among closely related species imply distinct evolutionary trajectories, which could be manifested at the genomic level. Morphological and behavioral investigations addressing the distinctive acoustic phenotype of the *macrotis* group further demonstrated that pinna and nasal capsule dimensions serve as better predictors of echolocation call frequencies than forearm length [29]. Studies using transcriptomics for *R. episcopus* and *R. siamensis* found differentially expressed genes relevant to the variation in echolocation frequency [40], and the expression variation related to acoustic signals (resting frequency) and body size (forearm length) was widely governed by natural selection [41]. However, little is known about the molecular basis on gene sequences for this specific phenomenon, which will help us to understand the evolution mode for the auditory system in bats.

Within the *macrotis* group, the standard body size–frequency allometric pattern is disrupted: most taxa emit frequencies lower than predicted by their body size (here termed species_low_), while *R. osgoodi* and the closely related *R. pusillus* conform to the conventional relationship (species_normal_) [34]. The molecular mechanisms behind this phenotypic divergence remain unclear. Transcriptomic approaches provide powerful opportunities to decipher the molecular genetic basis of adaptive evolution in the *macrotis* group. Here, we conducted comparative transcriptomic analyses using multi-tissue sequencing data generated from low-frequency and normal-frequency species within the *macrotis* group. We aimed to: (1) detect positively selected genes and significantly enriched Gene Ontology (GO) terms associated with adaptive evolution; (2) characterize sequence divergence between species_low_ and species_normal_; and (3) reveal the potential genetic mechanisms facilitating adaptation in species_low_.

## 2. Materials and Methods

### 2.1. Specimen Collection and Library Preparation

Species sequenced in this study included five species within the *macrotis* group and one closely related species *R. pusillus* (Table 1). One individual was sampled per species across four provinces of China, and three tissues (brain, liver and cochlea) were collected from each individual for transcriptome sequencing. Samples of the brain, liver, and cochlear were biopsied quickly after euthanasia, placed in RNA later, and then stored at −80 °C until RNA extraction. To avoid the influence of sex, we chose mature females and ensured none were pregnant. The animal study protocol was approved by the Laboratory Animal Welfare and Ethics Committee of Northeast Normal University under the protocol code NENU–20211015 on 15 October 2021.

Total RNA from these tissues was extracted using TRIzol reagent (Invitrogen Corp., Carlsbad, CA, USA). RNA purification was performed using a RNeasy Mini Kit (Qiagen, Chatsworth, CA, USA). Library constructions were made from the brain, liver, and cochlear of each species according to the Illumina Hiseq 2500 RNA sample preparation kit (Illumina, San Diego, CA, USA). We constructed two paired-end libraries with insert sizes of 150 base pairs (bp). All these original data have been deposited into the NCBI Sequence Read Archive database (Accession Number: SAMN35883750-SAMN35883755) (Table A1).

### 2.2. De Novo Assembly and Annotation of Transcripts

To explore genomic variation across the *macrotis* group, we generated the de novo transcriptome assembly for species sequenced. We obtained clean reads from raw data by removing reads containing adapter, reads containing ploy-N, and low-quality reads. All the downstream analyses were based on clean data. Transcriptome assembly was accomplished based on the pooled paired-end reads from three tissues using Trinity [42] with min_kmer_cov set to 2 and all other parameters set to default. We selected the longest transcript of a gene as the unigene and used it in the following analyses.

To obtain functional annotation for more unigenes, we used the genome data of *R. sinicus* and *H. armiger* from NCBI as references. First, the protein of each unigene was aligned to the NCBI Non-redundant (Nr) protein database using diamond v0.8.22 to produce annotation results. NCBI blast 2.2.28+ was then used to retrieve NCBI nucleotide sequences (Nt) for each unigene. Functional annotation of the unigene was undertaken based on the best match derived from the alignments to the proteins annotated in SwissProt and euKaryotic Ortholog Groups (KOG) database. And we used the HMMER 3.0 Package to annotate the unigenes in Protein family (Pfam). Descriptions of gene proteins from Gene Ontology (GO) ID were retrieved based on the results of NR and Pfam. Finally, the orthology of each protein was assigned by the Kyoto Encyclopedia of Genes and Genomes (KEGG) via the KAAS-KEGG Automatic Annotation Server based on the bi-directional best hit (BBH) method.

### 2.3. Identification of One-to-One Orthologous Gene Sets

We predicted the coding sequences (CDSs) of each unigene according to Nr and Swissprot. And we extracted the longest open reading frame (ORF) in the longest transcript per gene. Estscan 3.0.3 software [43] was also used to determine the direction of sequences that did not have aligned results, the CDSs extracted from these unigenes were translated into amino acid sequences with the standard codon table. We used OrthoMCL v2.0.3 [44] (e-value = 1 × 10^−3^) to identify orthologous genes using a Markov Cluster algorithm (MCL). The longest protein sequences per gene were used as the one-to-one orthologous genes among eight species in this study and analyzed in downstream analyses.

### 2.4. Phylogenetic Analysis

Before adaptive evolution analysis, we need a phylogenetic tree to measure the relationship among these species. Protein sequences of eight species were aligned using MUSCLE v3.8.31 [45]. After multiple sequence alignments and trimming by the program Gblocks, all one-to-one orthologous genes were concatenated to one sequence for each species and used to construct a phylogenetic tree. PhyML v3.1 [46] was applied to build a maximum likelihood (ML) phylogeny with 1000 bootstrap replicates, and we used FigTree version 1.4.2 (http://tree.bio.ed.ac.uk/software/figtree/, accessed on 13 May 2021) to visualize the topology.

### 2.5. Calculation of Evolution Rates

We aligned nucleotide sequences using MUSCLE for each orthologous gene and formed a concatenated alignment for all orthologs. We used the constructed tree topology as the guide tree, and calculated pairwise rates of dS, dN, and dN/dS using the free-ratio model (Parameters: model = 1, NSsites = 0, fix_omega = 0, omega = 1) in PAML 4 [47] for each branch. The dN/dS value was used to measure the evolutionary rate along a lineage. The lineage-specific mean value was estimated by concatenated alignments from all orthologs.

### 2.6. Positive Selection Analyses

We indicated positive selection by the ratio of the rate of nonsynonymous-to-synonymous substitutions (dN/dS or ω) per gene among lineages. Using our phylogenetic tree topology as the guide tree, we ran a branch-site model [47] (Parameters: null hypothesis: model = 2, NSsites = 2, fix_omega = 1, omega = 1; alternative hypothesis: model = 2, NSsites-2, fix_omega = 0, omega = 1) in the codeml module of PAML4 to detect the positive selection for the one-to-one orthologous genes of five species within the *macrotis* group, respectively. For each analysis, we selected only one species as a foreground branch, and regarded all other species as the background branches. A likelihood rate test (LRT) was performed to detect a positive selection on the foreground branch, and the Bayes Empirical Bayes (BEB) analysis [48] was implemented to calculate posterior probabilities and to deduce positively selected sites. *p*-values of all positively selected genes (PSGs) were also normalized by FDR using the Benjamini–Hochberg approach [49] and a *p* value less than 0.05 was considered significant.

### 2.7. Gene Ontology Enrichment Analyses

Gene Ontology (GO) was described with GO terms. GO enrichment analysis was implemented by GOseq 1.10.0 in R packages for the PSGs orthologous groups [50]. We analyzed the GO enrichment for five species of the *macrotis* group, respectively. GO categories were identified at three levels (biological process, molecular function, and cellular component) that were over-represented by PSGs. Enrichment *p*-values were calculated and all *p*-values were multi-normalized FDR using the best-hit approach. Categories with an FDR of less than 0.05 were considered over-represented.

## 3. Results

### 3.1. De Novo Transcriptome Assembly and Functional Annotation

Raw reads generated from RNA-sequencing for each of the three tissues (brain, liver, and cochlea) from *R. episcopus*, *R. siamensis*, *R. rex*, *R. marshalli*, *R. osgoodi*, and *R. pusillus* (Table 1) ranged from 44.617 Mb to 64.037 Mb (Table A2). After quality control, about 44.127 Mb (6.62 Gb) to 63.363 Mb (9.5 Gb) clean reads of three tissues remained for the de novo assembly (Table A2). The clean reads of three tissues were pooled for each species. We obtained the transcript for each species and extracted the unigene for the following analyses. For the unigene of each species, the longest nucleotide length is 199.489 Mb for *R. episcopus*, and the shortest is 138.272 Mb for *R. osgoodi*. The contig N50s parameters of all samples generally ranged from 914 bp to 1173 bp (Table A3).

GO terms for each of the annotated genes mainly covering biological GO categories at three ontologies levels (biological process, molecular function, and cellular component) were identified (Figure S1). The GO terms are similar for these species, and the biological processes annotated more unigenes. Our analyses of GO terms generally represented the main biological GO classification and ensured the integrity of the downstream functional analyses of the candidate genes.

### 3.2. Orthologous Gene Identification and Phylogenetic Tree

**Figure 2:**
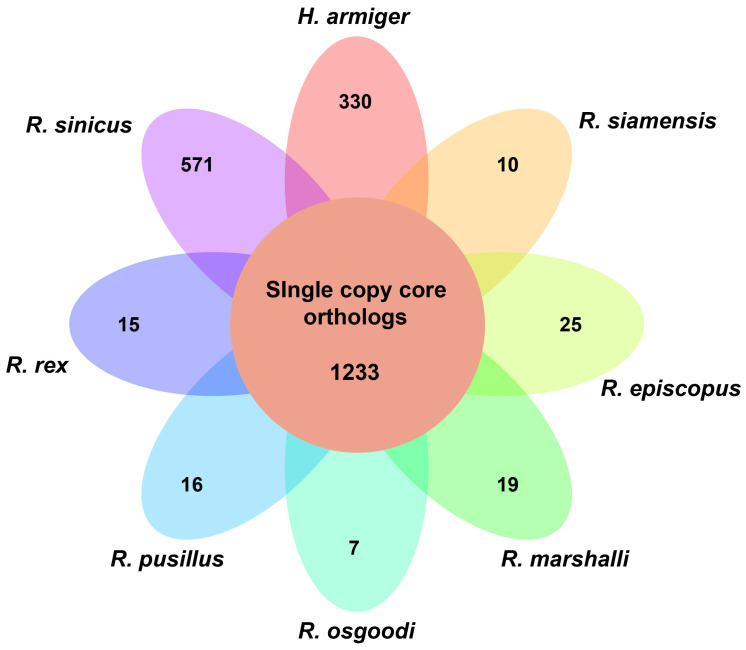
Venn diagram of orthologs genes for eight species.

We identified one-to-one orthologous gene pairs across six species sequenced (*R. episcopus*, *R. siamensis*, *R. rex*, *R. marshalli*, *R. osgoodi*, and *R. pusillus*) and two reference genomes (*R. sinicus* and *H. armiger*). In total, 1233 one-to-one genes were retained for the downstream analyses (Figure 2). Our final dataset, therefore, included eight species and 1233 genes.

**Figure 3:**
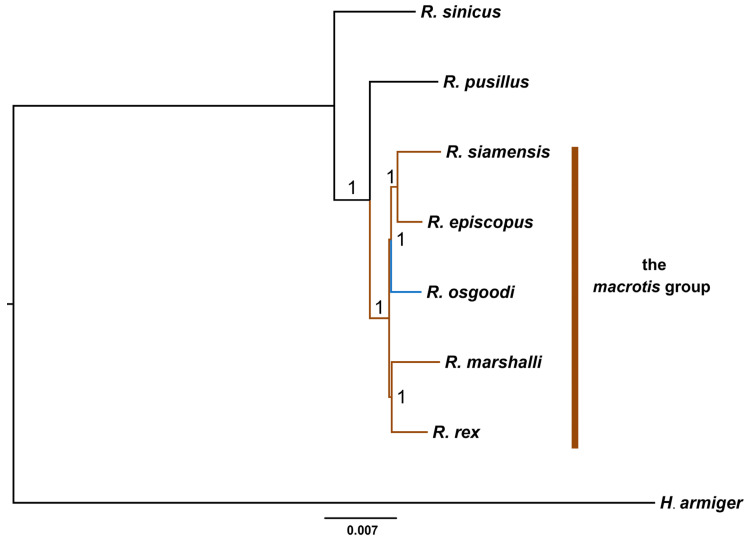
Maximum-likelihood tree constructed based on 1233 one-to-one single-copy ortholog genes of eight species. Colored branches indicate bat species within the *macrotis* group. Orange and blue lines represent species with low and normal echolocation frequency relative to body size respectively. The numbers represent the posterior probabilities of each clade.

High ML bootstrap values in our constructed phylogenetic tree have well-resolved phylogenetic relationships between species of the *macrotis* group and other species (Figure 3). *Hipposideros armiger* behaved as an outgroup in the topological relationship, and all rhinolophids clustered in one clade. Species of the *macrotis* group clustered together, and were closely related to *R. pusillus*. Among the *macrotis* group, the *R. macrotis* complex (*R. episcopus*, *R. siamensis* and *R. osgoodi*) clustered in one highly supported clade, and formed a paraphyly with the clade of *R. marshalli* and *R. rex*. The topology of our tree is congruent with previous phylogenies based on the mitochondrial genome [46].

### 3.3. Evolution Rate

**Figure 4:**
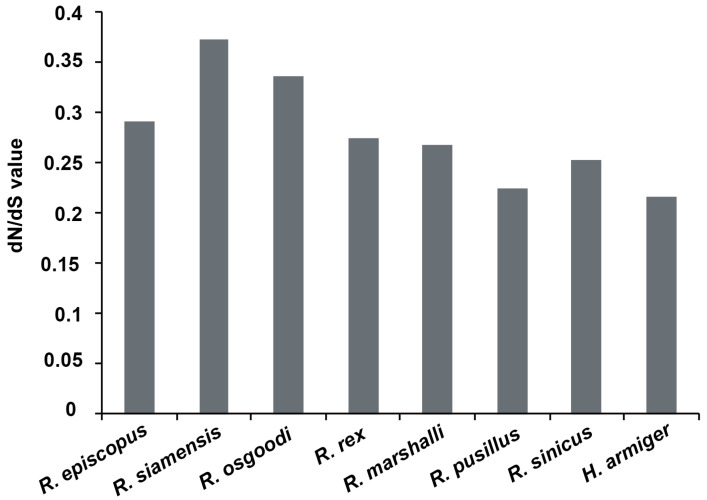
dN/dS values for each species calculated from sequence of concatenated homologous genes.

Because outlier genes with larger dN/dS may generate deviations in evaluating the overall selective constraint for species, we filtered our dataset to remove genes with dN/dS > 100. The rest of genes were used to evaluate the overall selective constraints in the species analyzed. The dN/dS value was calculated for each species in this study (Figure 4). The dN/dS ratios varied across the examined taxa: two species within the *macrotis* group, *R. siamensis* (0.3723) and *R. osgoodi* (0.3355), exhibited relatively higher dN/dS values (*R. episcopus*: 0.2919, *R. rex*: 0.2742, *R. marshalli*: 0.2683) compared with outgroup species (*R. pusillus*: 0.2250, *R. sinicus*: 0.2524, *H. armiger*: 0.2170). Such patterns hint that accelerated sequence evolution may occur in certain members of the *macrotis* group, though we caution that interpretation is limited by the use of only one individual per species.

### 3.4. Positive Selection in the Macrotis Group Lineage

**Figure 5:**
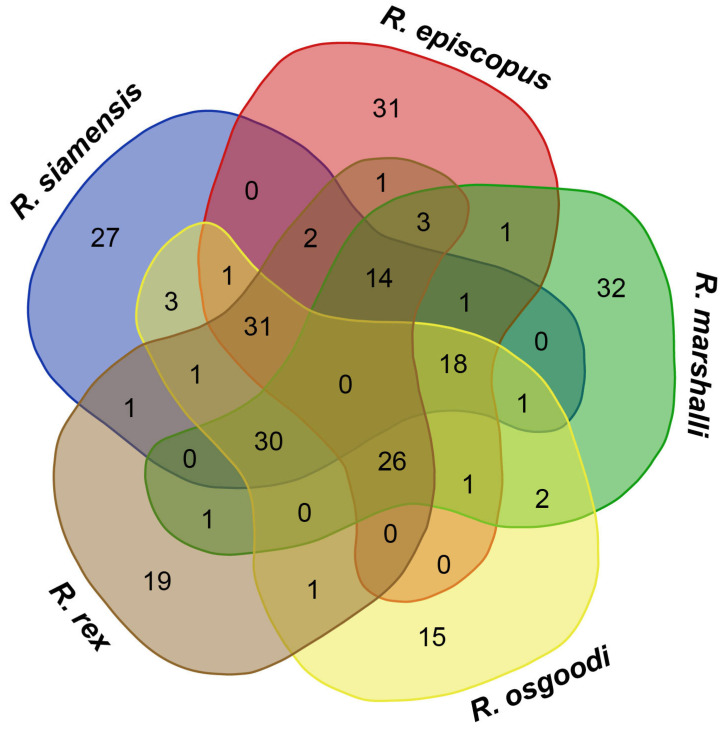
Venn diagram of positively selected genes selected from branches of species within the *macrotis* group. The numbers represent the number of species-specific or shared genes.

**Figure 6:**
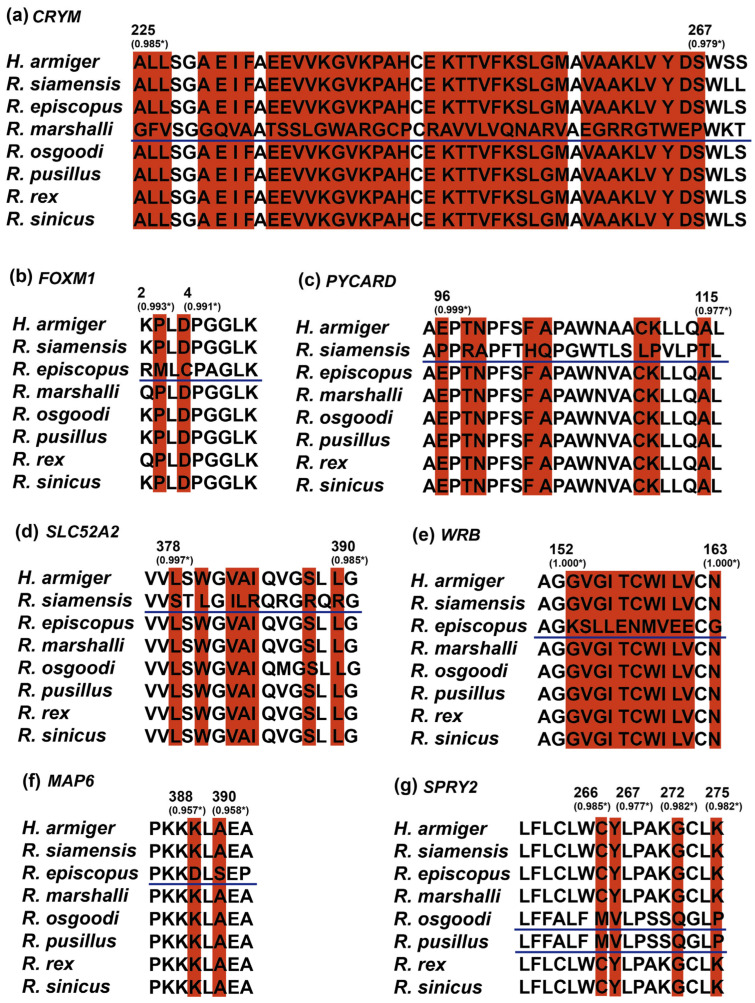
A part of amino acid sequence alignments of the genes related to hearing. (**a**–**f**) In these sequences, the first and the last positively selected sites were labeled, and the posterior probability of corresponding sites was represented. The species used to test the amino acid change are indicated by a line. (**g**) The positive selected sites are labeled and the posterior probability of corresponding sites is represented. The same amino acid change that occurred in *R. osgoodi* and *R. pusillus* is indicated by a line. Horizontal lines indicate that positively selected sites were detected in the corresponding species. Red color indicates the screened positively selected sites and asterisks (*) denote sites under significant positive selection.

A total of 141 candidate PSGs and 1274 positively selected sites were identified with signals of positive selection along each species branch of the *macrotis* group based on the branch-site model in PAML 4 (Figure 5, Table 2). Here, seven hearing-related genes (*CRYM*, *FOXM1*, *PYCARD*, *SLC52A2*, *WRB*, *MAP6*, and *SPRY2*) (Table 3) showed significant evidence of positive selection in species of the *macrotis* group. And the positively selected sites tested in species_low_ were species-specific and distinct from other species (Figure 5 and Figure 6a–f). This result may represent different adaptive responses of auditory development for species_low_. In particular, we found one hearing-related gene, *SPRY2*, was positively selected in *R. osgoodi*, which emits normal echolocation frequency in the *macrotis* group. We found the same amino acid mutation sites in four positively selected sites in *SPRY2* for *R. osgoodi* and *R. pusillus*, which were different from other species (Figure 6g). The daily activities for bats mainly rely on acoustics, but five visually related PSGs (*ARRDC3*, *LZTFL1*, *RAB8A*, *EGFBPL1*, and *TRNT1*) were tested in species_low_ (Table 4). No visual-related PSG was tested in species_normal_.

### 3.5. Functional Enrichment of PSGs

GO enrichment analyses showed that the candidate PSGs were significantly overrepresented and enriched into GO categories such as: metabolic process (GO: 0008152), protein metabolic process (GO: 0019538), and single-organism process (GO: 0044699) (Table 5). These categories appeared to be biologically relevant to energy metabolism.

By comparing the GO annotations enriched in different species_low_, we found some GO terms enriched more PSGs in each species. For the PSGs in *R. siamensis*, we listed six over-represented GO categories, and some of them related to catalytic activity and metabolic processes. For *R. episcopus*, three significantly enriched GO categories in terms of single-organism process, catalytic activity, and single-organism process enriched more PSGs. We found three GO categories enriched more PSGs compared with other categories in *R. marshalli*. The PSGs identified in *R. rex* were mainly enriched in GO terms of molecular function, single-organism process, catalytic activity, and protein metabolic process.

## 4. Discussion

Comparative transcriptomics has been widely applied to unravel the molecular basis of phenotypic adaptation [5,51,52,53]. Comparative analysis of auditory traits help clarify the genetic underpinnings of divergent auditory performance [26]. Within the *macrotis* group, pronounced acoustic divergence has been documented: most species produce echolocation frequencies lower than predicted by body size (low-frequency species), whereas *R. osgoodi* and the closely related *R. pusillus* follow typical allometric scaling (normal-frequency species). Here, we sequenced multi-tissue transcriptomes from five species of the *macrotis* group and *R. pusillus*, identifying key genes linked to adaptation in *Rhinolophus*. Our results indicate that species within the *macrotis* group evolved a suite of physiological adaptations.

We identified signatures of Darwinian selection in genes associated with hearing, vision, and energy metabolism, which may have driven the emergence of species-specific echolocation frequencies. Positive selection acting on hearing-related genes in rhinolophids is probably associated with the sophisticated auditory processing supporting CF echolocation.

### 4.1. Adaptive Mechanism of Hearing-Related Genes

Rhinolophids arguably possess the most sophisticated echolocation systems, and can emit relatively long calls adapted to detect and classify the wing beats of insects. They are heavily reliant on hearing for a variety of ecologically important roles. Previous studies have documented that hearing-related genes are predominantly evolutionarily conserved in mammals [54]. Comparative analysis of auditory perception can help to elucidate the molecular basis that underpins different auditory capabilities [26]. Dong et al. (2016) [26] revealed some hearing-related genes underwent natural selection associated with the evolution of specialized constant frequency echolocation, probably resulting from the extreme selectivity used in the auditory processing by these bats. We performed a comparative transcriptome analysis for species with two echolocation modes, and tested some genes under selective pressure for the species within the *macrotis* group.

We selected some specific amino acid changes in six PSGs (*CRYM*, *FOXM1*, *MAP6*, *PYCARD*, *SLC35A2*, and *WRB*) related to hearing. *CRYM* was tested in *R. marshalli*, and the previous functional study found mutations in *CRYM* may cause hearing loss and the expression of *CRYM* is essential for maintaining cochlear cells and preserving normal hearing [55,56]. We tested *FOXM1*, *MAP6*, and *WRB* in *R. episcopus*. Previous studies showed that *FOXM1* activities are modulated in the mouse cochlea, and *FOXM1* is associated with cell cycle control and is essential for the transcriptional response during DNA damage/checkpoint signaling [57]. *MAP6* is involved in molecular transport, nervous system development, and function, and is related to auditory fear conditioning [58]. The absence of *WRB* from inner hair cells results in significantly reduced intracellular levels of otoferlin, thus causing hair cell synaptic disruption and hearing impairment [59]. *PYCARD* and *SLC52A2* were tested in *R. siamensis*. Thereinto, *PYCARD* was related to hearing-loss and tinnitus [60]. Mutation in *SLC52A2* was associated with spinocerebellar ataxia with blindness and deafness type 2 [61]. We tested different hearing-related genes in different species_low_, and this may imply these species adapted to the environment through different evolutionary mechanisms.

Particularly, we found that one PSG *SPRY2* in *R. osgoodi*, which emits echolocation with a normal frequency, differs from other species within the *macrotis* group. *SPRY2* is related to hearing loss and plays an important role in the regulation of endochondral bone formation, which may influence early inner ear development [62,63,64]. The positive selection sites detected in *SPRY2* showed similar amino acid changes with *R. pusillus*, but different ones with other species. This result indicates that *SPRY2* plays an important role in echolocation development, and may experience similar acoustic evolution in *R. pusillus*, thus proving *R. osgoodi* and *R. pusillus* evolved similar acoustic characteristics.

Although the function of specific amino acid variants in these genes is still unclear, our results still indicate the potentially important function in acoustic development. These genes may regulate the neural activity or the formation process of inner ear structure, and then affect the sensitivity of specific frequency, that is, affect the echolocation signal for these bats.

### 4.2. Adaptive Mechanism of Vision-Related Genes

We tested five vision-related PSGs in four species_low_. *ARRDC3*, *LZTFL1*, and *TRNT1* reveal important roles in the retina [65,66,67]. *RAB8A* plays a vital part in regulating outer segment protein composition and photoreceptor function [68]. And *IGFBPL1* is associated with the growth of retinal ganglion cell axons [69].

A previous study has suggested that the enlargement of one area of the brain might be associated with the reduction in the size of other brain areas [70]. The auditory cortex and the inferior colliculus are enlarged in volume in laryngeal echolocating bats, especially in rhinolophoid bats, whereas visual brain areas are relatively enlarged in Old World fruit bats [71]. Because of the extreme energetic demands imposed by neural processing, the trade-off has been proposed to be an investment in brain tissues. Dong et al. (2013) [11] showed more visual perception genes have become pseudogenes in rhinolophoid bats, and speculate that some visual perception genes may have undergone relaxed natural selection in echolocating bats. They also found some hearing-related genes underwent positive selection, and such concordance suggests that some genes are impacted by natural selection, which raised the possibility that changes in sensory genes will have direct consequences for those genes controlling for other sensory modalities, perhaps via trade-offs. We found no vision-related genes affected by positive selection in *R. osgoodi* emit echolocation with normal frequency. This finding supports the longstanding but weakly supported assumption that bats are experiencing a trade-off between vision and audition [11].

Positive selection of both acoustic and visual genes was detected in species_low_, indicating that there was no obvious inhibition of the evolution of acoustic-related genes and vision-related genes in these species, which were affected by natural selection and are different in other species. These comparative analyses provide important evidence for the adaptation of species_low_ to their special hearing mechanisms. Although laryngeal echolocation bats do not rely primarily on vision, the evolution of one sensory-related gene may have influenced the evolution of other senses. So we cannot rule out the role of these genes in the evolution of bat hearing until further functional analysis is performed, especially for species in the *macrotis* group, which has a low dominant frequency of echolocation.

### 4.3. Adaptive Mechanism of Metabolism-Related Genes

Through the analysis of GO terms enriched by positive selection genes, we found that positive selection genes were mainly significantly enriched in GO terms related to metabolism, and involved in various metabolic processes, for example: the oxidation-reduction process, lipid metabolism process, catabolic process, and biosynthesis process. The abundance of these metabolism-related positive selection genes in species_low_ indicates that these species have the stronger positive selection in energy metabolism or biosynthesis than other species.

Adaptation of energy metabolism plays a very important role in the evolutionary process of species. Bats can fly, which requires huge energy consumption [72], so genes related to energy metabolism play an important role in the evolutionary process of bats. Owing to the high energy output of aerobic respiration, which underpins energy provision, species may acquire greater energy supplies via modifications to mitochondrial function and redox pathways. Some positive selection genes and positive selection sites were detected in mitochondria [73], the main energy supply organelles, which is consistent with the results of positive selection detected in genes related to energy metabolism in this study. These results suggest that energy-related genes have undergone adaptive evolution in species_low_, and these species have a stronger selective role in energy metabolism and may improve some aspects of adaptability by regulating energy metabolism in the process of evolution. Notably, it remains unclear whether echolocation frequency directly shapes flight energy budgets at the genetic level. An alternative hypothesis is that low-frequency species possess specialized foraging behaviors that elevate flight energy expenditure.

Although GO terms enriched by positive selection genes are mostly related to metabolism, there are differences in GO terms enriched among different species, indicating that these species may experience different evolutionary processes, and corresponding genes may play potentially important roles in the evolutionary process of different species.

### 4.4. General Evolutionary Overview of Bat Echolocation

Echolocation represents one of the most successful and distinctive evolutionary innovations across Chiroptera, having diversified over more than 50 million years ago and reshaped sensory, cranial and metabolic traits in distinct bat lineages [17,74]. Two core echolocation modes have evolved independently within bats: broadband FM calls used by most Vespertilionoid, and specialized narrowband CF echolocation unique to Rhinolophidae and Hipposideridae [75,76]. CF echolocation constitutes a derived adaptive complex coupled with enlarged pinnae, elaborate noseleaves and remodeled cochlear structures, enabling Doppler-shift compensation to detect tiny fluttering insect prey—an adaptive toolkit absent in non-echolocating fruit bats and FM bats [29].

Two universal evolutionary trends have been repeatedly validated across phylogenomic and transcriptomic studies covering all bat families: first, auditory genes consistently undergo positive selection to tune cochlear sensitivity to species-specific ultrasonic frequencies [35,38]; second, a widespread sensory evolutionary trade-off exists between hearing and vision, whereby enhanced echolocation capacity correlates with relaxed purifying selection or pseudogenization of retinal genes in echolocating taxa [77,78]. Additionally, powered flight imposes extraordinary energetic demands unmatched by other mammalian locomotion modes; accordingly, genes governing mitochondrial oxidation, lipid catabolism and aerobic respiration show pervasive adaptive signatures in all bat clades, regardless of echolocation type or body mass [73].

Prior comparative genomic research mostly contrasts echolocation adaptation between highly divergent bat orders or focuses on intraspecific transcriptional variation within single horseshoe bat populations [26,27]. Few multi-species transcriptomic investigations have resolved lineage-specific molecular shifts underlying subtle inter-genus acoustic divergence, such as the disrupted body size–frequency allometry observed in the *macrotis* species group [29]. As a monophyletic assemblage of recently diverged Pleistocene taxa with divergent low/normal-frequency phenotypes, this clade provides a valuable microevolutionary model to fill this research gap. Our identification of divergent positively selected auditory, visual and metabolic genes within this group aligns with the overarching evolutionary framework of bat sensory adaptation, while refining our understanding of how lineage-specific selective pressures reshape coding sequences during recent cladogenesis.

## 5. Conclusions

In summary, our comparative transcriptome analyses of the brain, liver, and cochlear in the *macrotis* group provided the theoretical basis for the adaptive mechanism in the evolution of species_low_. The evidence of genetic adaptation provides insights into hearing-related genetic adaptation, vision-related genetic adaptation and energy metabolism-related genetic adaptation. And GO enrichment analysis revealed that PSGs detected in the species_low_ mainly enriched in GO terms related to energy metabolism. The important genes and mutations selected may be of great significance for bat adaptability. However, more detailed functional studies of all the selected candidate genes will be necessary to confirm the roles of individual genes. Studying the function and potential pathways of these genes will contribute to a better understanding of phenotypic evolution in bats, especially for the evolution of bat acoustic. Further studies of species_low_ will play an important role in understanding their adaptive evolution mechanisms. Beyond the lineage-specific adaptive signatures uncovered within the *macrotis* group, our multi-tissue transcriptomic results also expand the global evolutionary framework of bat echolocation evolution. The co-occurrence of positively selected sensory and metabolic genes in species_low_ recapitulates universal adaptive patterns observed across all echolocating bat lineages, while the unique breakdown of body size–frequency scaling in this species provides a valuable microevolutionary case study for future cross-family comparative analyses of chiropteran sensory adaptation.

## Figures and Tables

**Table 1:** Sampling information and sequenced tissues.

Species	Vouch Number	Collection Site	Sex ^a^	Brain ^b^	Liver ^b^	Cochlear ^b^
*R. episcopus*	HuN17009	Lengshuijiang, Hunan	F	√	√	√
*R. siamensis*	GX17032	Nanning, Guangxi	F	√	√	√
*R. rex*	GZ17016	Bijie, Guizhou	F	√	√	√
*R. marshalli*	GX17004	Nanning, Guangxi	F	√	√	√
*R. osgoodi*	YN17114	Lijiang, Yunnan	F	√	√	√
*R. pusillus*	GX17029	Nanning, Guangxi	F	√	√	√

^a^ F, Female. ^b^ “√” indicates that this tissue was sequenced.

**Table 2:** Branch-site likelihood ratio tests and corresponding number of positively selected genes and sites.

Test No.	Species	Number of Positively Selected Genes	Number of Positively Selected Sites
1	*R. episcopus*	34	190
2	*R. siamensis*	28	364
3	*R. osgoodi*	18	177
4	*R. rex*	24	217
5	*R. marshalli*	37	326
Total		141	1274

**Table 3:** Hearing-related genes under positive selection in the *macrotis* group.

Test Branch	Gene	Full Gene Name	Gene Function
*R. siamensis*	*SLC52A2*	Solute Carrier Family 52 Member 2	deafness
	*PYCARD*	PYD And CARD Domain Containing	cochlea
*R. episcopus*	*MAP6*	Microtubule Associated Protein 6	auditory
	*FOXM1*	Forkhead Box M1	nervous deafness
	*WRB*	tryptophan rich basic protein	hearing impairment
	*POLD1*	DNA Polymerase Delta 1	deafness
	*SLC35D1*	Solute Carrier Family 35 Member D1	chondrocyte development
*R. marshalli*	*POLD1*	DNA Polymerase Delta 1	deafness
	*CRYM*	Crystallin Mu	deafness
	*SLC35D1*	Solute Carrier Family 35 Member D1	chondrocyte development
*R. rex*	*SLC35D1*	Solute Carrier Family 35 Member D1	chondrocyte development
*R. osgoodi*	*SPRY2*	Sprouty RTK Signaling Antagonist 2	hearing loss

**Table 4:** Positively selected genes associated with vision tested from species of the *macrotis* group.

Test Branch	Gene	Full Gene Name	Gene Function
*R. siamensis*	*ARRDC3*	Arrestin Domain Containing 3	retina
	*LZTFL1*	Leucine Zipper Transcription Factor Like 1	retina
*R. episcopus*	*RAB8A*	Member RAS Oncogene Family	outer segment protein composition
*R. marshalli*	*IGFBPL1*	Insulin Like Growth Factor Binding Protein Like 1	the growth ofretinal ganglion cellaxons
*R. rex*	*TRNT1*	TRNA Nucleotidyl Transferase 1	retina

**Table 5:** GO enrichment analysis for the five species of the *macrotis* group (only the GO terms with the most positively selected genes in each species are listed).

Species	GO ID	GO Terms	Number of Positively Selected Genes	*p*-Values
*R. siamensis*	GO:0003674	molecular_function	18	1.29 × 10^−2^
	GO:0008152	metabolic process	14	1.35 × 10^−2^
	GO:0003824	catalytic activity	13	1.07 × 10^−2^
	GO:0044699	single-organism process	13	3.63 × 10^−4^
	GO:0044763	single-organism cellular process	10	5.46 × 10^−3^
	GO:0008150	biological_process	10	3.55 × 10^−2^
*R. episcopus*	GO:0044699	single-organism process	17	2.82 × 10^−3^
	GO:0003824	catalytic activity	14	4.93 × 10^−2^
	GO:0044763	single-organism cellular process	14	3.55 × 10^−2^
*R. marshalli*	GO:0044699	single-organism process	16	7.86 × 10^−3^
	GO:0044248	cellular catabolic process	3	7.86 × 10^−3^
	GO:0009056	catabolic process	3	4.44 × 10^−2^
*R. rex*	GO:0003674	molecular_function	13	3.12 × 10^−2^
	GO:0044699	single-organism process	13	1.49 × 10^−2^
	GO:0003824	catalytic activity	12	1.68 × 10^−2^
	GO:0019538	protein metabolic process	7	3.51 × 10^−2^

## Data Availability

We sequenced transcriptomes of the brain, liver, and cochlea for six species, including five within the *macrotis* group (*R. episcopus*, *R. siamensis*, *R. rex*, *R. marshalli*, and *R. osgoodi*) and one closely related species, *R. pusillus.* All these original data have been deposited into the NCBI Sequence Read Archive database (Accession Number: SAMN35883750-SAMN35883755).

## Supplementary Materials

The following supporting information can be downloaded at: https://www.mdpi.com/article/10.3390/ani16152331/s1, Figure S1: GO classification of six species sequenced.

## Abbreviations

The following abbreviations are used in this manuscript:

Nr	non-redundant
Nt	nucleotide sequences
KOG	euKaryotic Ortholog Groups
Pfam	Protein family
GO	Gene Ontology
KEGG	Kyoto Encyclopedia of Genes and Genomes
BBH	bi-directional best hit
CDS	coding sequences
ORF	open reading frame
MCL	Markov Cluster algorithm
ML	maximum likelihood
LRT	likelihood rate test
BEB	Bayes Empirical Bayes
PSGs	positively selected genes

## Appendix A

**Table A1:** Genebank accession number of the sequenced species in this study.

Species	Accession Number
*Rhinolophus episcopus*	SAMN35883750
*Rhinolophus siamensis*	SAMN35883751
*Rhinolophus osgoodi*	SAMN35883752
*Rhinolophus rex*	SAMN35883753
*Rhinolophus marshalli*	SAMN35883754
*Rhinolophus pusillus*	SAMN35883755

**Table A2:** Quality assessment information for transcriptome sequencing.

Species	Sample	Raw Reads	Clean Reads	Clean Bases	Error (%)	GC Content (%)
*R. marshalli*	GX17004N	54,349,388	53,726,488	8.06 G	0.03	50.31
*R. marshalli*	GX17004E	54,184,596	53,589,816	8.04 G	0.03	50.63
*R. marshalli*	GX17004G	56,616,678	56,011,742	8.4 G	0.02	49.19
*R. episcopus*	HuN1709N	58,745,442	58,100,532	8.72 G	0.03	50.1
*R. episcopus*	HuN1709G	61,550,412	60,992,424	9.15 G	0.02	49.83
*R. episcopus*	HuN1709E	44,617,302	44,126,514	6.62 G	0.03	50.97
*R. siamensis*	GX17032N	60,708,108	59,871,120	8.98 G	0.03	50.81
*R. siamensis*	GX17032G	52,951,608	52,383,792	7.86 G	0.02	49.98
*R. siamensis*	GX17032E	60,654,954	59,881,756	8.98 G	0.02	50.86
*R. pusillus*	GX17029N	55,824,430	55,107,628	8.27 G	0.03	51.01
*R. pusillus*	GX17029G	51,668,604	50,834,932	7.63 G	0.02	50.05
*R. pusillus*	GX17029E	57,084,796	56,268,792	8.44 G	0.02	51
*R. rex*	GZ17016N	64,036,568	63,362,630	9.5 G	0.03	50.56
*R. rex*	GZ17016G	56,462,730	55,907,604	8.39 G	0.02	50.69
*R. rex*	GZ17016E	56,465,924	55,805,334	8.37 G	0.02	50.75
*R. osgoodi*	YN17114N	45,707,412	45,217,288	6.78 G	0.03	49.63
*R. osgoodi*	YN17114G	47,658,664	47,046,750	7.06 G	0.03	49.86
*R. osgoodi*	YN17114E	53,690,326	52,849,574	7.93 G	0.03	50.4

**Table A3:** Assemble results of transcript and Unigene.

	Min Length	Mean Length	Median Length	Max Length	N50	N90	Total Nucleotides
Transcript							
*R. episcopus*	201	872	346	27,511	2261	280	289,683,829
*R. marshalli*	201	913	352	32,085	2439	288	342,926,243
*R. siamensis*	201	887	345	31,288	2360	281	329,781,807
*R. rex*	201	916	353	33,524	2450	289	295,931,053
*R. osgoodi*	201	940	362	29,010	2445	297	232,224,455
*R. pusillus*	201	867	345	29,032	2185	277	243,263,086
Unigene							
*R. episcopus*	201	616	315	27,511	919	252	174,367,170
*R. marshalli*	201	625	319	32,085	942	254	199,488,875
*R. siamensis*	201	615	315	31,288	914	252	195,594,955
*R. rex*	201	638	320	33,524	999	255	174,917,500
*R. osgoodi*	201	670	324	29,010	1173	257	138,271,944
*R. pusillus*	201	626	310	29,032	1003	251	146,004,000
