# Supplementary material for: Gene Ontology-Based Comparative Transcriptomics Provides Evidence of Genetic Adaptations in the Rhinolophus macrotis Group (Chiroptera: Rhinolophidae)
Source: Animals (Basel). 2026 Jul 30;16(15):2331. doi: 10.3390/ani16152331 (PMC13463596; doi:10.3390/ani16152331)
Supplement: Supplementary file 1 [file animals-16-02331-s001.zip › animals-4431087-supplementary.pdf]

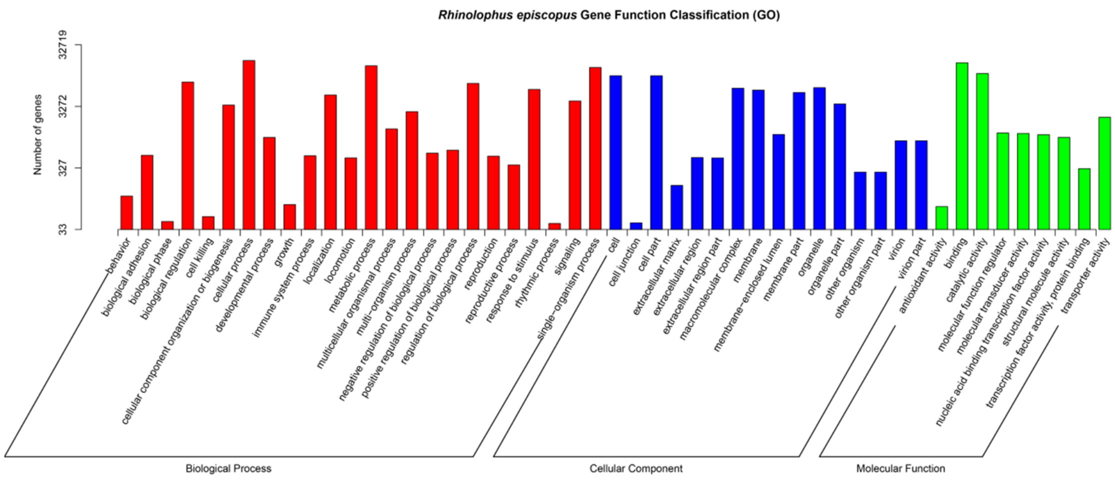

GO classification of *R. episcopus*

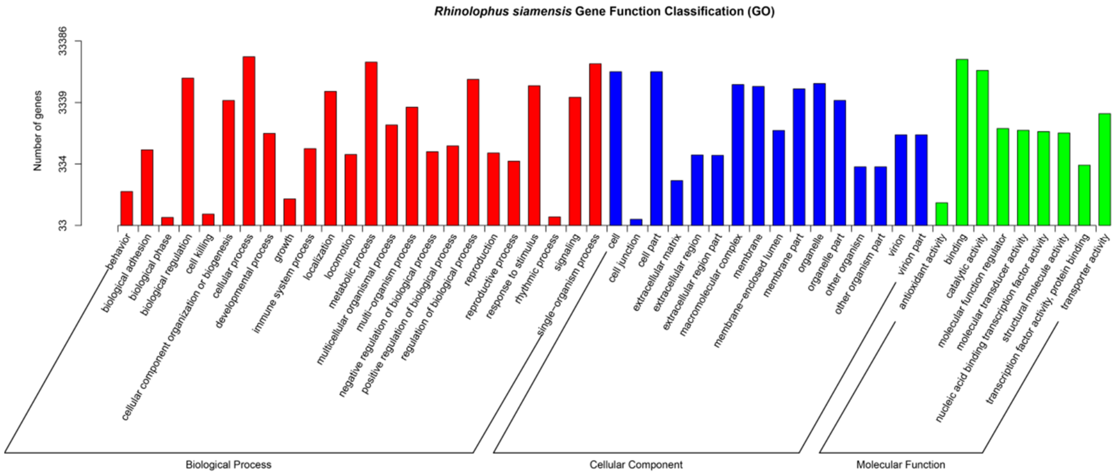

GO classification of *R. siamensis*

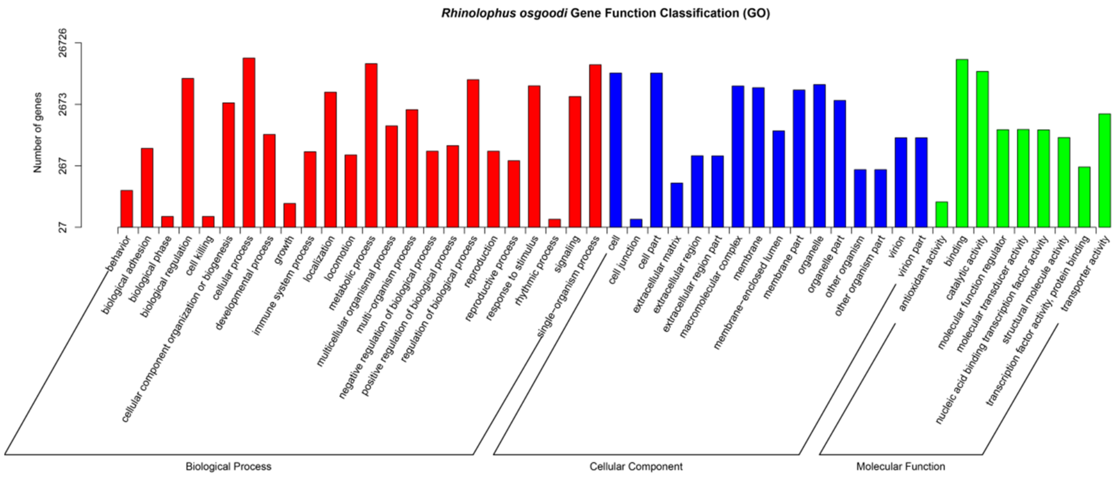

GO classification of *R. osgoodi*

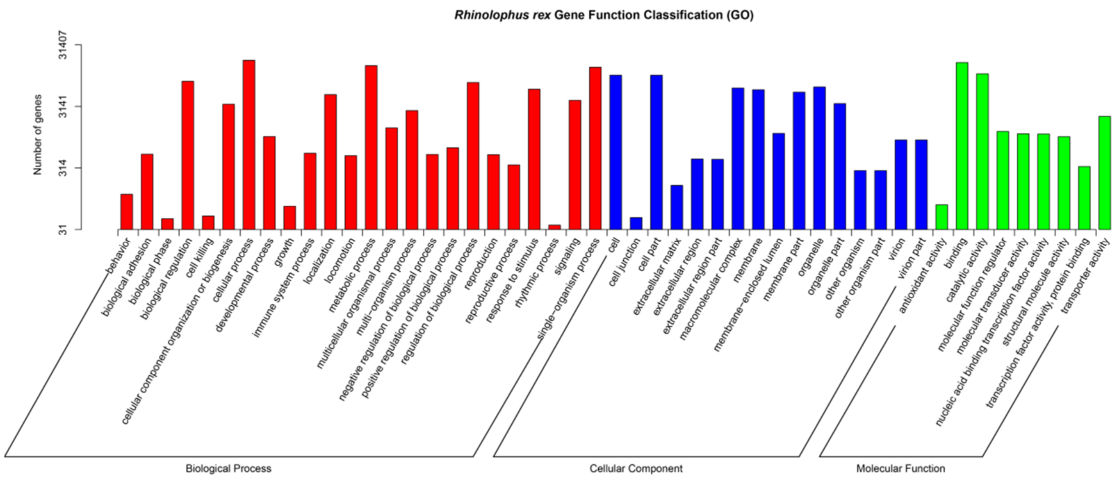

GO classification of *R. rex*

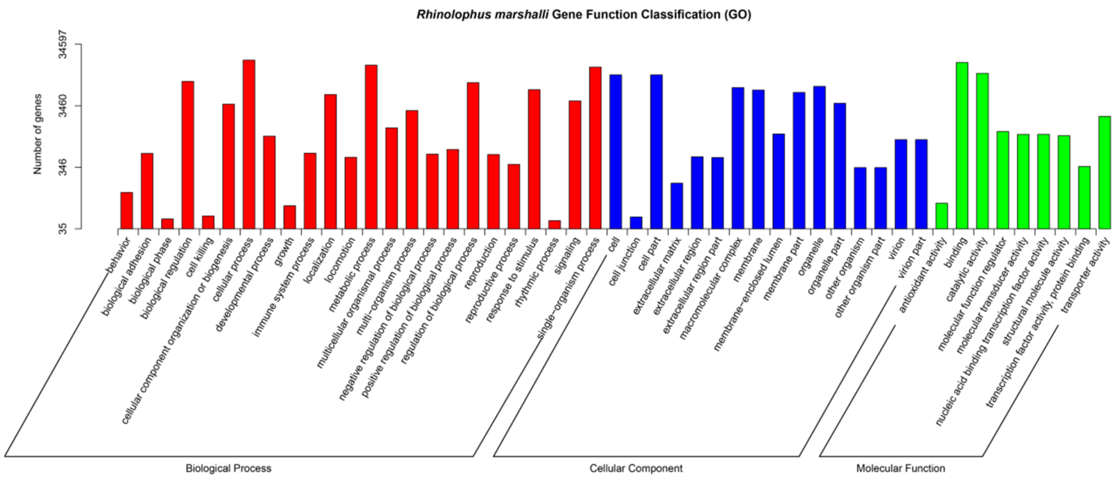

GO classification of *R. marshalli*

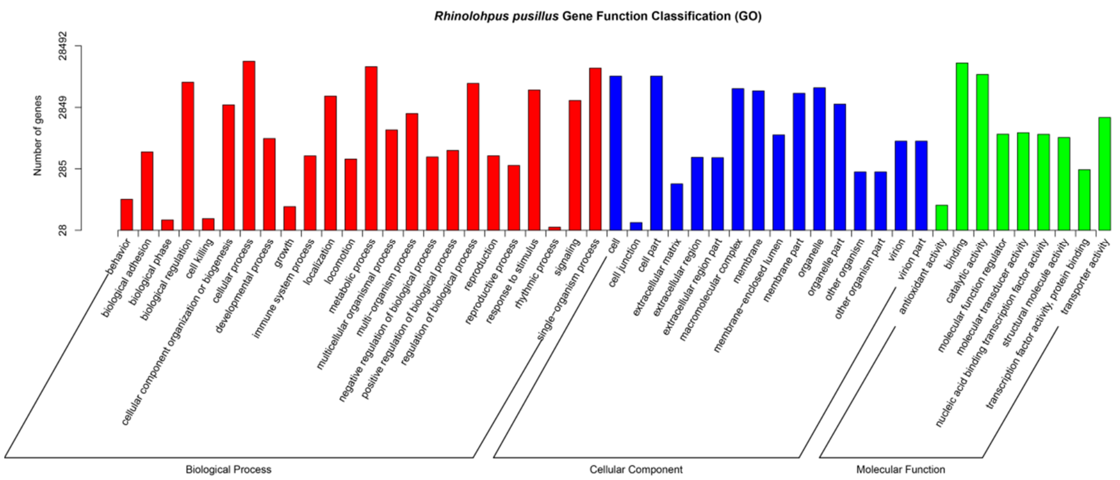

GO classification of *R. pusillus*

Figure S1: GO classification of six species sequenced.
